# Vitamin D levels and COVID 19 risk and death; is there an association?

**DOI:** 10.1186/s12937-022-00798-6

**Published:** 2022-06-24

**Authors:** Maisa Hamed Al Kiyumi

**Affiliations:** Department of Family Medicine and Public Health, Sultan Qaboos University Hospital, P.C.123, Al-Khod, P.O.Box 35, Muscat, Sultanate of Oman

Dear editor,

I read with great interest the study entitled “Low vitamin D levels do not aggravate COVID-19 risk or death, and vitamin D supplementation does not improve outcomes in hospitalized patients with COVID-19: a meta-analysis and GRADE assessment of cohort studies and RCTs” by Chen et al. [1]. This meta-analysis refuted any association between low vitamin D level and COVID-19 risk or death. However, I have the following comments related to the internal validity of this study:

1. The pooled analysis in Figure 2 revealed a nonsignificant association between vitamin D level and COVID-19 risk and death. However, it is obvious that the pooled effect size might be influenced by a study conducted by Hastie et al. [2, 3]. This study was based on participants recruited from the UK Biobank and did not show any association between low vitamin D level and the risk of developing COVID-19 after adjustment for potential confounders [2, 3]. Notably, the true prevalence of COVID-19 might be underestimated in this cohort because the PCR test results were available for only 1,474 participants out of 348,598 recruited [2]. Moreover, we need to interpret the findings of this study cautiously, as the baseline vitamin D level measurement was done more than a decade ago (2006–2020) [2]. Similarly, in another study by Katz et al. vitamin D measurements were performed during the preceding five years prior to the COVID 19 test [4]. Besides, it was not known whether those recruited participants with low vitamin D level received a treatment or not, which eventually might affect the accuracy of the data. I believe that carrying out sensitivity analysis in this case might help in mitigating the influence of these studies on the overall effect size.
2. Confounding factors and methods of measuring vitamin D level: While using the adjusted odds ratio plays a key role in attenuating the effects of the confounding variables, the studies included differ considerably on the types of confounding adjustment. For example, the included odds ratio by Kanz et al. [4] were adjusted for obesity only (OR = 2.27; 95% CI, 1.787–2.872; *P* < 0.001), whereas Kaufmann had adjusted for race/ethnicity, gender and latitude [5]. Also, with the exception of one study by Meltzer et al. [6], the possibility of vitamin D supplement initiation or adjustment after testing was not addressed and therefore might affect the findings of this meta-analysis. Importantly, the authors did not mention the assay used for measuring vitamin D level, which might also have impacted the accuracy of the overall findings. For instance, a study substantiates a higher concentration of vitamin D level when measured by a liquid chromatography-mass spectrometry technique (LC–MS/MS) compared to radioimmunoassay, with a mean difference of about 12.9 ng/ml [7].
3. In the statistical analysis section, the authors carried out the pooled analysis using a random effect model. In view of the few studies included in the quantitative analysis, the choice of which effect model to use (random versus fixed effects model) needs to be explained to the reader. While the random effects model can still be applied, the fixed effects model might be more suitable in this meta-analysis [8].

Considering the above factors, the results of this analysis need to be cautiously interpreted, and well-designed prospective clinical trials are necessary.

## Data Availability

Not applicable.
